# Draft Genome Sequence of the Archiascomycetous Yeast *Saitoella complicata*

**DOI:** 10.1128/genomeA.00220-15

**Published:** 2015-05-28

**Authors:** Kenta Yamauchi, Shinji Kondo, Makiko Hamamoto, Yurika Takahashi, Yoshitoshi Ogura, Tetsuya Hayashi, Hiromi Nishida

**Affiliations:** a Biotechnology Research Center and Department of Biotechnology, Toyama Prefectural University, Imizu, Toyama, Japan; b National Institute of Polar Research, Tokyo, Japan; c Department of Life Sciences, School of Agriculture, Meiji University, Kanagawa, Japan; d Frontier Science Research Center and School of Medicine, University of Miyazaki, Miyazaki, Japan

## Abstract

The draft genome sequence of the archiasomycetous yeast *Saitoella complicata* was determined. The assembly of newly and previously sequenced data sets resulted in 104 contigs (total of 14.1 Mbp; *N*_50_, 239 kbp). On the newly assembled genome, a total of 6,933 protein-coding sequences (7,119 transcripts, including alternative splicing forms) were identified.

## GENOME ANNOUNCEMENT

The subphylum *Taphrinomycotina* (*Archiascomycetes*) is the earliest ascomycetous lineage that diverged before the separation of the subphyla *Pezizomycotina* (*Euascomycetes*, filamentous ascomycetes) and *Saccharomycotina* (*Hemiascomycetes*, budding ascomycetous yeasts) (1, 2). The anamorphic and saprobic budding yeast *Saitoella complicata* is a member of the *Taphrinomycotina*, which was isolated from Himalayan soil (3). Interestingly, *S. complicata* shares some characteristics with both ascomycetous and basidiomycetous yeasts (3, 4).

We previously attempted to assemble the genome sequence of *S. complicata* using 454 (Roche) sequences (5) and Illumina paired-end read pairs (6). Although these previous assemblies were of a large number of small contigs, at 7,981 contigs (13.0 Mbp) (5) and 1,800 contigs (14.2 Mbp) (6), respectively, we found that the amino acid sequences of protein-coding genes identified on the contigs showed the highest similarity to proteins of *Pezizomycotina* (5, 6).

To elucidate the detailed characteristics of the *S. complicata* genomic DNA sequences, we have refined the genome assembly with additional sequencing of mate-paired DNA libraries of this species. We generated a total of 11.4 million paired-end read pairs (700-bp insert and 100 bp in length) and a total of 23.7 million mate-paired read pairs (6.2 million 3-kb-, 6.2 million 5-kb-, 5.3 million 10-kb-, and 6.0 million 15-kb-long-insert read pairs), respectively, using Illumina HiSeq and MiSeq sequencers. The read pairs were dereplicated by Fulcrum (7) and assembled using the SPAdes assembler (8). The assembly of the dereplicated read pairs by using 21 to 89 bp for the *k*-mer size option yielded a set of 104 contigs of ≥1 kb, whose total size and *N*_50_ are 14.1 Mb and 239 kb, respectively.

Using Augustus (9), a gene prediction software based on the alignment of expressed sequences to the genome, we have determined coding sequences (CDSs) of the genes expressed on the assembled genome of *Saitoella* according to the gene model of *Aspergillus nidulans*, which is thought to have some taxonomic proximity to *Saitoella*. Based on the exon coordinates mapped by a total of 89.3 million RNA sequencing (RNA-seq) paired-end read pairs (100 bp in length) uniquely mapped to the genome by BLAT (10), Augustus identified 6,933 protein-coding genes (7,119 transcripts, including alternative splicing forms) on the *Saitoella* genome. All this computational work was done on the NIG Supercomputer system (11).

### Nucleotide sequence accession numbers. 

The DNA sequences have been deposited in DDBJ under the accession numbers BACD03000001 to BACD03000104.
